# A New Essay on Man

**Published:** 1894-12

**Authors:** 


### Selections and Abstracts.

A New Essay on Man—With Apologies to Pope.—Man that is born of woman is small potatoes and few in a hill. He riseth up to-day and flourishes like a rag-weed, and to-morrow or the next day, the undertaker hath him. He goes forth in the morning warbling like a lark and is knocked out in one round and two seconds.

In the midst of life he is in debt, and the tax collector pursueth him wherever he goeth. The banister of life is full of splinters, and he slides down with considerable rapidity. Woe be to the seat of his understanding. He walketh forth in the bright sunlight to absorb ozone, and meeteth the bank teller with a sight draft for $357.

He cometh home at eventide and meeteth the wheelbarrow in his path. It riseth up and smitheth him to the earth and falleth upon him and runneth one of its legs into his ear.

He buyeth a watch-dog, and when he cometh home from the lodge the watch-dog treeth him and sitteth near him until rosy morn. He goeth to the horse trot and betteth his money on the brown mare, and the bay gelding with a blaze face winneth.

He marrieth a red-headed heiress with a wart on her nose, and the next day the parent ancestor goeth under with a fee, arrest and great liabilities, and cometh home to live with his beloved son-in-law.—*Ex.*

